# Microencapsulation and the Characterization of Polyherbal Formulation (PHF) Rich in Natural Polyphenolic Compounds

**DOI:** 10.3390/nu10070843

**Published:** 2018-06-28

**Authors:** Syed Ammar Hussain, Ahsan Hameed, Yusuf Nazir, Tahira Naz, Yang Wu, Hafiz Ansar Rasul Suleria, Yuanda Song

**Affiliations:** 1 Colin Ratledge Center for Microbial Lipids, School of Agriculture Engineering and Food Science, Shandong University of Technology, Zibo 255049, China; ammarshah88@yahoo.com (S.A.H); ahsanhameed@outlook.com (A.H.); yusufnazir91@yahoo.com (Y.N.); nazkhan658@gmail.com (T.N); neverhangsome@hotmail.com (Y.W.); 2 UQ Diamantina Institute, Translational Research Institute, Faculty of Medicine, The University of Queensland, 37 Kent Street Woolloongabba, Brisbane, QLD 4102, Australia; 3 Department of Food, Nutrition, Dietetics and Health, Kansas State University, Manhattan, KS 66506, USA; 4 Centre for Chemistry and Biotechnology, School of Life and Environmental Sciences, Deakin University, Pigdons Road, Waurn Ponds, VIC 3216, Australia

**Keywords:** microencapsulation, polyphenols, freeze-drying, antioxidant activity, in vitro dialyzability, in vitro anti-diabetic potential

## Abstract

Microencapsulation of polyherbal formulation (PHF) extract was carried out by freeze drying method, by employing gum arabic (GA), gelatin (GE), and maltodextrin (MD) with their designated different combinations as encapsulating wall materials. Antioxidant components (i.e., total phenolic contents (TPC), total flavonoids contents (TFC), and total condensed tannins (TCT)), antioxidant activity (i.e., DPPH, β-carotene & ABTS^+^ assays), moisture contents, water activity (a_w_), solubility, hygroscopicity, glass transition temperature (T_g_), particle size, morphology, in vitroα-amylase and α-glucosidase inhibition and bioavailability ratios of the powders were investigated. Amongst all encapsulated products, T_B_ (5% GA & 5% MD) and T_C_ (10% GA) have proven to be the best treatments with respect to the highest preservation of antioxidant components. These treatments also exhibited higher antioxidant potential by DPPH and β-carotene assays and noteworthy for an ABTS^+^ assays. Moreover, the aforesaid treatments also demonstrated lower moisture content, a_w_, particle size and higher solubility, hygroscopicity and glass transition temperature (T_g_). All freeze dried samples showed irregular (asymmetrical) microcrystalline structures. Furthermore, T_B_ and T_C_ also illustrated the highest in vitro anti-diabetic potential due to great potency for inhibiting α-amylase and α-glucosidase activities. In the perspective of bioavailability, T_A_, T_B_ and T_C_ demonstrated the excellent bioavailability ratios (%). Furthermore, the photochemical profiling of ethanolic extract of PHF was also revealed to find out the bioactive compounds.

## 1. Introduction

In the past decade, research has been focused in exploring naturally occurring antioxidants to circumvent the multifaceted health related complexities arising due to overproduction of reactive oxygen species (ROSs) in body [1,2]. The excess production of these ROSs are considered serious issue for human health as their surplus generation can lead to different patho-physiological conditions like fast aging process via damaging the nucleic acids and changing in the conformation of proteins, heart-related disorders (i.e., cardiovascular disorders (CVDs)), diverse types of cancers, immunity related dysfunctions, inflammation, membranous lipid oxidation, decline of hydroperoxide synthesis, neurodegenerative disorders, lungs and kidney illness, UV-irradiation, and osteoporosis/bone-related diseases and health-related disease called “oxidative stress” [3]. In addition, direct correlation between oxidative stress and insulin resistance (key factor for type-II DM) has also been elaborated in mini review by Hurrle et al. [4].

Amongst naturally-occurring antioxidants, polyphenols and their derivative compounds represented a diverse class of ubiquitous material i.e., from simple molecules to complex configuration such as phenolic acids; hydroxybenzoic and hydroxycinnamic acids, hydrolyzable and condensed tannins, and flavonoids, these are most important compounds for nutraceutical, therapeutics and pharmacological point of view [5,6] and revealed various health endorsing activities: antioxidant activity (i.e., free radicals scavenging, declining of hydroperoxide development, hampering the lipid oxidation), anti-diabetic, anti-malarial, and anticancer activity etc. [5,7].

In the prospective of natural polyphenols, polyherbal formulations are considered as a great source all over the globe due to their dynamic medicinal and therapeutic claims. Moreover, previous investigations illustrated that selected individual plants contained abundant quantity of polyphenols and their herbal combinations were found to produce best antioxidant activity among all individual extracts due to synergistic effect. Synergism played a vital role via two different kind of mechanism in context of interaction i.e., pharmacokinetic (PK) and pharmacodynamics (PD) [8,9]. Owing to synergism, polyherbal formulation demonstrated vast advantages over single herbal formulation (SHF) likewise: superior restorative effect can be attained with a PHF; to acquire enviable pharmacological accomplishment low dosage would be required, consequently lessening the risk of harmful side effects. Additionally, PHF facilitate the patient’s convenience by eradicating the need of taking more than one formulation at a time, which ultimately leads to better compliance and therapeutic effect. All the aforesaid advantages have outcome in the attractiveness of PHF in the marketplace when compare to SHF [10].

Polyphenols are incredibly sensitive in diverse range of circumstances, during food processing and storage practice likewise; high temperature of surrounding, incidence of oxygen and light, pH, existence of oxidative enzymes, moisture contents [11]. The degradation of natural antioxidants may hamper the possible effectiveness of application of these antioxidants in food/nutraceutical and pharmaceutical applications and commercially available anti-diabetic drugs also produce unconstructive effects on other metabolisms [12], so supplementation of anti-hyperglycemic substances, which also possess antioxidant properties, might be an alternative therapy to overcome this critical condition [13,14]. To address these shortcomings and to augment the antioxidant stability and preserve their diverse bioactivities including anti-inflammatory, anti-cancer, anti-microbial, anti-diabetic capabilities, the microencapsulation has been employed successfully as a reliable technique to circumvent the unwanted degradation of bioactive compounds, shielding them from adverse environmental circumstances. Furthermore, various type of wall material has been used for microencapsulation procedure, but cost effectiveness and physico-chemical distinctiveness must be considered, including: hygroscopicity, biodegradability, emulsifying feature, adaptability to gastrointestinal tract (GT), viscosity, solids content [15].

At present, the preferred wall materials for microencapsulation for various fruit juices and plant/herbs extracts are maltodextrins (MD), gum arabic (GA) and gelatin (GE) [16]. Maltodextrin of various dextrose equivalents (DE) are generally used as wall material owing to their distinct characteristics likewise; low viscosity, high solubility in water and their solutions are monochromic in appearances. These features made them frequently used carrier/wall materials in the micro-encapsulation procedure. Gum arabic (exudates of acacia), owing to its unique features i.e., naturally colorless, low viscosity, high retention of volatiles and ability to make stable emulsion is ultimately considered as excellent encapsulating agent whereas its high economic cost provoked researcher for full or partial replacement of the encapsulation agent [16,17,18]. In addition, gelatin is also a better option for microencapsulation because of its superior characteristics for emulsification, film-formation, water solubility, last but not least ability to form finer dense complex. According to Fang and Bhandari [19], a sole microencapsulating agent has limitation over all required attributes to improve microencapsulation effectiveness, eventually has been resolved by using different combination of polymers due to their diverse features. The selection for polymer’s combinations which possibly consequence in superior microencapsulating efficiency and regarded economically suitable than the single biopolymers has been becoming the point of emerging interest [19,20].

In the current study, polyherbal formulation was firstly made with equal ratio of roots of *Chlorophytum borivilianum*, roots of *Astragalus membranaceu*s, roots of *Eurycoma longifolia*, and seeds of *Hygrophila spinosa* T. Anders having previously proven diverse ethno-pharmacological applications [21,22,23,24] as polyphenols enriched nutrient supplement, then PHF extract was further microencapsulated by freeze drying method using different wall materials, subsequently antioxidant components (i.e., TPC, TFC, and TCT), antioxidant activity (i.e., DPPH, β-carotene & ABTS^+^ assays), anti-diabetic potential (i.e., in vitro α-amylase and α-glucosidase inhibition), physical properties like; moisture contents, water activity (a_w_), solubility, hygroscopicity, glass transition temperature (T_g_), morphological characteristics (i.e., particle size, morphology), and bioavailability ratios of the microencapsulated powders were investigated. In last, the chemo-profiling for ethanolic extract of PHF was also studied.

## 2. Materials and Methods

### 2.1. Materials, Chemicals, Reagents and Encapsulating Agents

All different parts of herbs (detail in Section 2.2) were purchased from Faisalabad-Pakistan and their identification and respective characteristics were authenticated by Prof. M. Jafar Jaskani from Institute of Horticulture, University of Agriculture Faisalabad (UAF) Pakistan. All chemicals used were of analytical grade or higher where suitable. DPPH (2,2-diphenyl-1-picryl-hydrazyl), Folin-Ciocalteu (FC),β-carotene, Butylated hydroxyltoluene (BHT), TWEEN 20, quercetin, Sodium carbonate,ABTS (2,20-azinobis (3-ethylbenzothiazoline-6-sulphonic acid), α-tocopherol, Linoleic acid, (+)-catachin, quercetin, AlCl_3_·6H_2_O, HCl, Vanilline, NaOH, Potassium persulfate,Trolox, gallic acid were purchased from Sigma-Aldrich GmbH (Sternheim, Germany). α-amylase fromporcine pancreas,α-glucosidase from *Saccharomyces cerevisiae*, paranitrophenyl-glucopyranoside, pepsin (porcine-7000), bile salts pancreatin (p-1750), piperazine-*NN*-bis (2-ethane-sulfonic acid) di-sodium salt (PIPES), gelatin (GE), HPLC-grade methanol, acetonitrile ethanol, acetone were supplied by Sigma-Aldrich (St. Louis, MO, USA), soluble starch (extra pure) was obtained from J. T. Baker Inc. (Phillipsburg, NJ, USA). Ultra-pure water (18 MΩ cm^−1^) was acquired from Milli–Q purification device (Millipore Co., Billerica, MA, USA). Sodium hydrogen carbonate was purchased from Merck (Darmstadt, Germany). Sea sand was of 200–300 grain size from Scharlau (Barcelona, Spain). The encapsulating agents were: gum arabic (Sangon Biotech Co., Shanghai, China), maltodextrin (Dextrose equivalent of 12) was purchased from Corn Products (Cabo de Santo Agostinho, Pernambuco, Brazil).

### 2.2. Polyherbal Formulation

Polyherbal formulation was made by combining the root of *Chlorophytum borivilianum,* roots of *Astragalus membranaceus*, roots of *Eurycoma longifolia*, and seeds of *Hygrophila spinosa* T. Anders, in a ratio of 1:1:1:1 respectively.

### 2.3. Preparation of Sample

Firstly, the roots and seeds of aforesaid herbs were cut into small pieces, followed by thorough washing with deionized water in order to avoid any contamination. The PHF material was then dried for 12 days in dark in well ventilated room at room temperature (23 ± 8 °C), and subsequently grounded with mortar and pestle to make crude powder with the help of liquid nitrogen, until a uniform sieve size equivalent to (1.0 mm) was achieved. The resulting powder was stored at −80 °C in inert vacuum bags until used for extraction as followed.

### 2.4. Pressurized Liquid Extraction (PLEx)

PLEx was executed in a Dionex ASE 350 system (Dionex, Sunnyvale, CA, USA) with the powder of PHF obtained as mentioned above. Aliquot of 5.0 g of powder of PHF was mixed with diatomaceous earth (1/1) and placed in a 34 mL stainless–steel cells. The extraction was performed via 3 consecutively applied steps with absolute solvents of increasing polarity, in order to get the maximum possible number and amount of secondary metabolites of various polarities and miscibilities, namely, acetone, ethanol, methanol and their aqueous mixtures with water (1:10, 3:10), and pure water. Extraction time was of 22 min; pressure 10.6 MPa; temperature 75 °C (for acetone, ethanol and methanol) and 135 °C (for water). Organic solvents were removed in a rotary vacuum evaporator at 38 °C, while the residual water was removed in a freeze drying unit. The extracts after solvent evaporation were placed under nitrogen flow for 20 min and stored in dark glass bottles at −80 °C until analyzed.

### 2.5. Development of Microencapsulated Powder Products

In order to prepare the particular dispersions, 100 mL of water was mixed with aforesaid PHF extract individually with different preselected combination of microencapsulating wall materials as follow: A (5% GA & 5% GE) (hereafter referred and discussed as T_A_); B (5% GA & 5% MD) (hereafter referred and discussed as T_B_), C (10% of GA)(hereafter referred and discussed as T_C_), and D (10% of MD) (hereafter referred and discussed as T_D_),under constant shaking with 220 rpm, at 35 °C for 30 min by a shaking unit (CIMO instrument manufacturing Co., Shanghai, China). Afterward, these dispersions were microencapsulated through lyophilization process for formulating four distinctive treatments. i.e., T_A_, T_B_, T_C_, T_D_.

For microencapsulation by means of freeze-drying process, the above-mentioned dispersions/emulsions were kept at −20 °C (freezer) for 48 h. Subsequently, the samples were placed in lyophilization unit (Labconco, Kansas, MO, USA) for freeze drying at −56.5 °C, with vacuum pressure of 4.61 mmHg for 60 h. After the completion of freeze drying process, the samples were crushed utilizing a mortar and pestle assembly. Finally, the desirable final microencapsulated products were sealed in polyethylene bags and aluminum pouches as well and stored in desiccator encompassing silica until further analysis.

### 2.6. Determination of Bioactive Compounds and Their Bioactivities after Microencapsulation

Bioactive components which were determined after the microencapsulation were total phenolic compounds (TPC), total flavonoids compounds (TFC), total condensed tannins (TCT). While the bioactivities of the microencapsulated powders were measured in terms of total antioxidant activity determined by β-carotene bleaching assay (TOAA), ABTS^+^ radical scavenging activity, and DPPH scavenging capacity. All these spectrophotometric analysis were performed according to previously developed methods with minor alteration [2,25,26]. The results for ABTS^+^ radical scavenging activity are deliberated as EC_50_ values (mg of extract/mL) for comparison. Effectiveness of antioxidant properties is inversely correlated with EC_50_ value.

### 2.7. Determination of the Physical Properties of the Microencapsulated Powders

#### 2.7.1. Moisture Content

The moisture contents of the microencapsulated products were estimated by using the method describes in manual AOAC [27], i.e., by calculating the loss of sample after weight after heat up at 105 °C.

#### 2.7.2. Water Activity (a_w_)

The water activity (a_w_) of all lyophilized samples was calculated through the direct analysis in electronic meter (Aqualab 3TE-Decagon, Pullman, WA, USA), to gain the constant state the samples were firstly placed at 25 °C for at least 15 min.

#### 2.7.3. Solubility

The solubility of microencapsulated products was measured by the method described by Cano-Chauca et al. [28], with minute alterations. The sample’s quantity of 1.0 g was mixed up with 100 mL distilled water in beaker and stirred with magnetic stirrer (MS-H-S10) for 20 min. After that the centrifugation of solution carried out at 3000× *g* (Thermo Scientific, Waltham, MA, USA) for 10 min. The quantity of 25 mL of the supernatant was transferred to a petriplates (pre-weighted) and dried in oven at 105 °C for 4.0 h. The solubility was measured as a result of weight difference and demonstrated in the term of percentage (%).

#### 2.7.4. Hygroscopicity

For the estimation of hygroscopicity, the microencapsulated powders of 1.0 g were placed in dessicator with saturated NaCl solution (74.6%) at temperature of 25 °C. After 1 week, samples were weighed and hygroscopicity were calculated in the term of percentage (%) [29].

#### 2.7.5. Glass Transition Temperature (T_g_)

The glass transition temperature (T_g_) of the microencapsulated products was calculated by means of differential scanning calorimetry (DSC) (DSC-2000-New Castle, DE, USA). The weight of 7–8 mg of sample was placed in aluminum hermetic pots. For the reference purpose, an aluminum pan without sample was used. Ultra-pure nitrogen N_2_ was used as purge gas (flow rate 50 mL/min). The temperature ranged from −80 °C to 120 °C at a heating rate of 40 °C/min. The glass transition temperature was determined by utilizing software of TA Universal Analysis 2000.

### 2.8. Morphology and Size Distribution

The configuration of micro-particles obtained from diverse encapsulating wall material and their combinations were examined by scanning electron microscope (Quanta 250 EFI). At first, very minute was fixed on surface of double sided tape of carbon then finally evaluated the samples under microscope with 400× magnification. The analysis for particle size distribution average and particle size was conducted by the means of ImageJ (NIH, Bethesda, MD, USA).

### 2.9. In Vitro Assays

#### 2.9.1. α-Amylase Inhibition Assay

The inhibition of α-amylase was determined using an assay modified from the Worthington Enzyme Manual [30]. Aliquot 0–4 mg/mL in DMSO (*v*/*v* 1:1) of each encapsulated PHF samples was prepared and 500 μL of each sample were mixed with 500 μL of 0.02 M sodium phosphate buffer (pH 6.9) containing α-amylase solution (0.5 mg/mL) and incubated at 25 °C for 10 min. After pre-incubation, 500 μL of a 1% starch solution in 0.02 M sodium phosphate buffer (pH 6.9) was added to each tube at timed intervals. The reaction mixtures were then incubated at 25 °C for 10 min. The reaction was stopped with 1.0 mL of dinitrosalicylic acid color reagent. The test tubes were then incubated in a boiling water bath for 5 min and cooled to room temperature. The reaction mixture was then diluted by adding 15 mL of distilled water, and the absorbance was measured at 540 nm using a micro-plate reader (Thermomax, Molecular Device Co., Sunnyvale, CA, USA). The experiments were performed in duplicate and the absorbance of sample blanks (buffer instead of enzyme solution) and a control (buffer in place of sample extract) were also recorded. The absorbance of the final each encapsulated PHF sample was obtained by subtracting its corresponding sample blank reading. Acarbose was prepared in distilled water and used as positive controls.

The percentage inhibition was calculated using the formula;

% Inhibition = {(Ac − Ae)/Ac} 100

where Ac and Ae are the absorbance of the control and extract, respectively.

IC_50_ values (inhibitor concentration at which 50% inhibition of the enzyme activity occurs) of each encapsulated PHF samples were determined by plotting graph with varying concentrations of the plant extracts against the percent inhibition.

#### 2.9.2. α-Glucosidase Inhibition Assay

The α-glucosidase was assayed using a method modified by Apostolidis et al. [31]. Aliquot of 0–4 mg/mL in DMSO (*v*/*v* 1:1) of each encapsulated PHF samples were prepared. 50 μL of each concentration sample was mixed well with 100 μL of 0.1 M phosphate buffer (pH 6.9) containing α-glucosidase solution (1.0 U/mL) and the mixtures were then incubated in 96-well plates at 25 °C for 10 min. After pre-incubation, 50 μL of 5 mM p-nitrophenyl-α-d-glucopyranoside solution in 0.1 M phosphate buffer (pH 6.9) was added to each well at timed intervals. The reaction mixtures were incubated at 25 °C for 5 min. Before and after incubation absorbance readings were recorded at 405 nm using a micro-plate reader (Thermomax, Molecular Device Co.) and compared to a control which contained 50 μL of the buffer solution instead of the extracts. The experiments were performed in triplicate and the α-glucosidase inhibitory activity was expressed as percentage inhibition. Acarbose was prepared in distilled water and used as positive controls. The percentage inhibition was calculated using the formula;

% Inhibition = {(Ac − Ae)/Ac} 100

where Ac and Ae are the absorbance of the control and extract respectively.

IC_50_ values (inhibitor concentration at which 50% inhibition of the enzyme activity occurs) of each encapsulated PHF samples were determined by plotting graph with varying concentrations of the plant extracts against the percent inhibition.

#### 2.9.3. Determination of Bioavailability of Microencapsulated Products by In Vitro Dialyzability Assay

The estimation for bioavailability of all microencapsulated products was determined by the method developed by Pineiro et al. [32].

### 2.10. Acute Toxicity

The acute oral toxicity study was carried out in compliance with Organization for Economic Cooperation and Development (OECD) guideline 425 [33]. All mice (*n =* 5) for testing were fasted for 12 h and weigh have been recorded and subsequently received the solution of microencapsulated products of PHF at the final concentration of 2000 mg/kg by gavage. The animals were observed individually at least once during the first 30 min after dosing, periodically for first 24 h and regularly thereafter for 14-day of feeding period for gross behavioral changes, toxicity symptoms or mortality.

### 2.11. LC-ESI-QTOF-MS Analyses

For LC-ESI-QTOF-MS analysis, firstly ethanolic extract was prepared using PLEx as described in Section 2.4. Afterwards obtained ethanolic extract was used to for the metabolite profiling of PHF using an Agilent 1100 Liquid Chromatography system (Agilent Technologies, Palo Alto, CA, USA) furnished with a standard auto-sampler. The analytical column used was characterized as Phenomenex Gemini C18 (3 μm, 2 × 150 mm) operated at 25 °C with a gradient elution portfolio at a flow rate of 0.2 mL/min. The mobile phases used were of acidified water (0.5% acetic acid) (A) and acetonitrile (B). The following multi-step linear gradient applied in following fashion: 0 min, 5% B; 5 min, 15% B; 25 min, 30% B; 35 min, 95% B; 40 min, 5% B. The initial conditions were maintained for 5 min. The injection volume of sample in system was 1μL. The LC-MS system was further composed of a Dionex Ultimate 3000 Rapid Separation LC system coupled to a micrOTOF QII mass spectrometer (Bruker Daltonics, Bremen, Germany) fitted with an electro-spray source operating in positive mode. The LC system contained an SRD-3400 solvent rack/degasser, an HPR-3400RS binary pump, a WPS-3000RS thermostated auto-sampler, and a TCC-3000RS thermostated column compartment. The micrOTOF QII source parameters were as follows: temperature, 200 °C; drying N_2_ flow, 8 L/min; nebulizer N_2_, 4.0 bar; end plate offset, −500 V; capillary voltage, −4000 V; mass range, 50−1500 Da, acquired at 2 scans/s. Post acquisition internal mass calibration used sodium formate clusters with the sodium formate delivered by a syringe pump at the start of each chromatographic analysis. Nitrogen was used as drying, nebulizing and collision gas. The precise mass data of the molecular ions were processed using Data Analysis 4.0 software (Bruker Daltoniks, Bremen, Germany), which delivered a list of potential elemental formulas via the Generate Molecular Formula Editor. The generate molecular formula Editor uses a CHNO algorithm, which deals with standard practicalities such as electron configuration, minimum/maximum elemental range and ring-plus double-bond equivalents, as well as a sophisticated comparison of the theoretical with the measured isotope pattern (Sigma Value, Bruker Daltonics, Bremen, Germany) for increased confidence in the recommended molecular formula. The commonly acknowledged accuracy threshold for validation of elemental compositions was established at 5 ppm [34]. It is significant to point out that even with very high mass precision (<1 ppm) many chemically likely formulas may be found, subjected to the mass regions considered and so high mass accuracy alone is not enough to discount enough candidates with complex elemental compositions. The use of isotopic abundance patterns as a single further constraint, however, eliminates >95% of the false candidates. This orthogonal filter can diminish numerous thousand nominees down to only a small number of molecular formulas. During the development of the HPLC method, the instrument was calibrated externally with a 74900-00-05 Cole Palmer syringe pump (Vernon Hills, Chicago, IL, USA) directly linked to the interface and injected with a sodium acetate cluster solution containing 5 mM sodium hydroxide and 0.2% acetic acid in water: isopropanol (1:1, *v*/*v*). The calibration solution was injected at the beginning of each run and all the spectra were calibrated prior to compound identification. By using this method, an exact calibration curve based on several cluster masses, each differing by 82 Da (NaC_2_H_3_O_2_) was obtained. Due to the compensation of temperature drift in the micrOTOF-Q II, this external calibration provided accurate mass values of better than 5 ppm for a complete run without the need for a dual sprayer setup for internal mass calibration.

## 3. Statistical Analysis

All statistical analyses were conducted using a one-way analysis of variance using Dunnett’s comparison tests or unpaired t-tests. All calculations were carried out using GraphPad Prism 5 (GraphPad Software, San Diego, CA, USA, www.graphpad.com). Significance was observed at *p* < 0.05.

## 4. Results and Discussion

### 4.1. The Effect of Microencapsulation on the Contents of Antioxidant Components and Antioxidant Activity of PHF

The contents of antioxidant components of PHF extract treated with different encapsulating wall materials were shown in Table 1. In comparison to untreated extract, all microencapsulated treatments have less antioxidant components (i.e., TPC, TFC & TCT). The retention for all freeze-dried treatments demonstrated in the term of percentages, ranged from 94.28% to 68.22% for TPC, 76.46% to 40.35% for TFC and 79.24% to 59.70% for TCT representing the effectiveness of microencapsulation procedure. The wide-ranging powders produced from the microencapsulation process, especially those obtained from T_C_, retained higher contents of antioxidant components. In general, these results may be associated with the type and concentrations of different wall materials. There were many multifaceted factors which were responsible for hammering of polyphenol compounds during freeze drying method, the crushing of lyophilized microencapsulated products after freeze-drying, were considered one of the key factors which may cause the degradation of bioactive components in the final products by boosting the product’s contact with environment. Our finding was in agreement with previous work in which authors explored that lyophilized wine product contained almost 70% of the original phenolics components [35,36]. Other factors which may responsible for declining the concentration of active components include: formation of microspheres during the lyophilization due to a scattering of the bioactive components inside the configuration of encapsulating wall materials i.e., consisting of one or more constant phase of encapsulating agents [19], development of micro-pores in the aforesaid microspheres, mainly associated to sublimation process during lyophilization [37]. In the current study, lyophilized product encompassed a reduction of 5.72–31.78% for TPC, declined trend of 23.54–59.65% and 20.76–40.30% was also observed for TFC and TCT respectively. Despite the reduction of antioxidant components of microencapsulated products, a significant retentions were also observed (described above in detail with percentages) comparable/higher to prior studies i.e., authors found, that acai pulp microencapsulated with GA have phenolic retention of 94.1% [16].

The freeze dried product microencapsulated with 10% GA (T_c_) demonstrated the exceptional conservation for antioxidant components (i.e., TPC, TFC & TCT). The order of effectiveness of microencapsulation for other remaining treatments was as followed: T_B_ > T_A_ > T_D_. The higher competence of T_C_ treatment was mainly attributed to the structure of GA, because it is a hetero-polymer made up of dense branches of sugar, containing a minute quantity of protein which connected to the carbohydrate skeleton via covalent bonds, proceeding as a tremendous microencapsulating material [38]. Noteworthy results were also found for T_B_ and T_A_, which might be credited to presence of 5% GA. In contrary, no significant difference was noticed for the lyophilized product having10% MD as wall material (T_D_).

The antioxidant activity for microencapsulated powders determined by DPPH, β-carotene and ABTS^+^ assay were illustrated in Table 1. All microencapsulated products had showed decrease antioxidant by DPPH assay in relation to original extract (control) and their retention ranged from 38.84–64.50%. T_B_ (5% GA & 5% MD) and T_c_ (10% GA) illustrated the highest antioxidant activity; these results were agreement with previously found values by Souza et al. [39]. The order for effectiveness was noticed as: T_B_ > T_C_ > T_A_ > T_D_. In the case of β-carotene bleaching assay, the antioxidant retention for all microencapsulated products were explored from 77.59% to 93.93% in comparison to original extract, T_B_ (5% GA & 5% MD) showed maximum value for antioxidant activity in a similar way as in DPPH assay. Remaining treatments have been categorized in context of efficacy as followed: T_C_ > T_D_ > T_A_. Referring to antioxidant assay by ABTS^+^ radical scavenging activity, the range of retention was from 62.2% to 86.68%. The noteworthy consequence was revealed for T_A_ (5% GA & 5% GE), while T_B_ (5% GA & 5% MD) and T_C_ (10% GA) also illustrated the significant results with retention of 75.27% and 74.18% respectively. The above discussion suggested the worthiness of diverse antioxidant assay for secure and overwhelming conclusion, because each assay comprised its own preciseness and proceeds at a challenging site of action. Amongst the all lyophilized encapsulated products, the antioxidant activity was higher in T_B_ and T_C_, being related to the presence of high antioxidant components (i.e., TPC, TFC & TCT) (Table 1), which provided an excellent defense system against unrestrained oxidation, owing to its high reducing power. Furthermore, there is no report yet on microencapsulation of aforesaid polyphenol enriched extract from PHF and their characterization related to analysis for antioxidant.

### 4.2. Physical Characteristics of Microencapsulated Powder Products

Physical factors i.e., water activity; moisture contents and hygroscopicity are indispensable for encapsulating products steadiness and storage, whilst aqueous solubility is correlated with ability of powder products for reconstitution [18].

**Figure 1:**
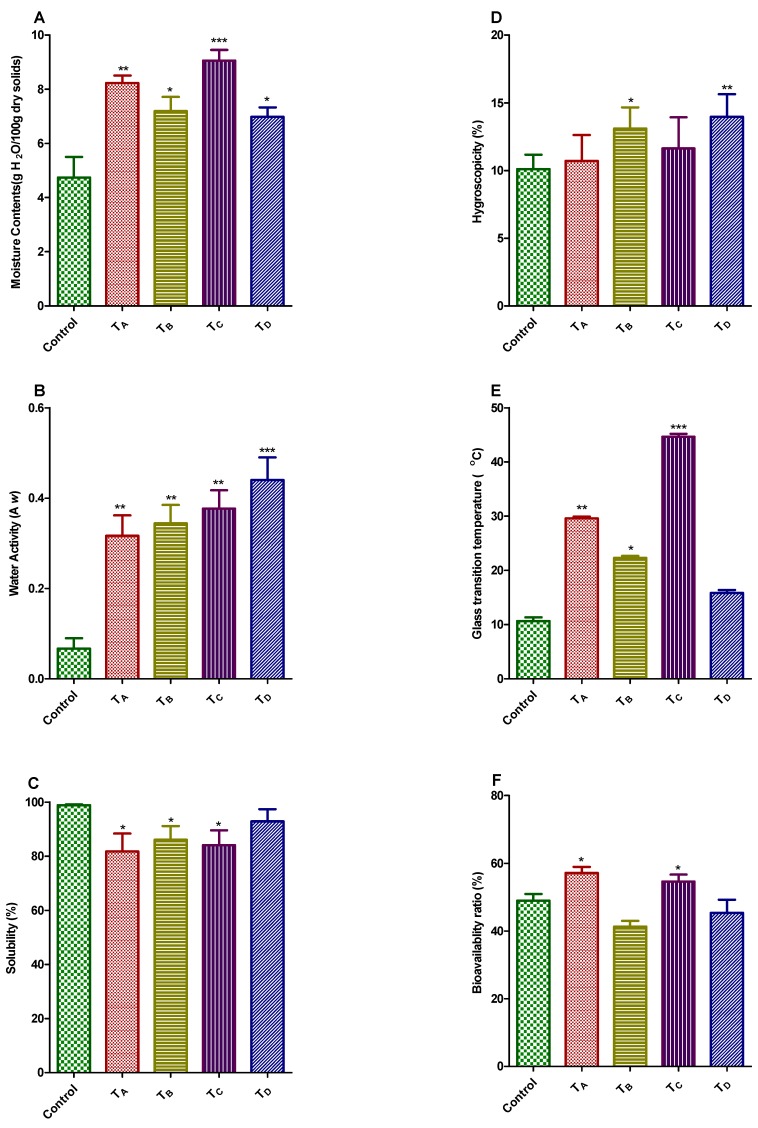
(**A**–**F**) Physical properties and bioavailability ratio (%) of PHF extract microencapsulated with GA, GE, MD and their combinations by freeze-drying method. Treatment A (T_A_): Freeze-dried, with 5% GA & 5% GE; Treatment B (T_B_): Freeze-dried, with 5% GA & 5% MD; Treatment C (T_C_): Freeze-dried, with 10% GA; Treatment D (T_D_): Freeze-dried, with 10% MD.

The moisture contents for four different lyophilized encapsulated products were demonstrated in Figure 1A. The moisture content of said powders were ranged from 7.07% to 9.04%; on the contrary, no significant difference was found between T_B_ and T_D_ (7.41% and 7.21%, respectively). Our findings was validated by earlier investigation which elaborated the moisture contents for blackberry fruit drink encapsulated by means of MD and trehalose dehydrate were of 2.44–6.11% [40]. Lower freezing temperature i.e., less than −40 °C consequences in quick freezing, eventually caused tiny pores in the superficial coatings, which might encumber the mass transfer and regarded as an obstacle for sublimation process, causing the higher retention of moisture contents in microencapsulated products [41].

The water activity (a_w_) of all microencapsulated products (Figure 1B) was ranged from 0.310 to 0.450, and all final encapsulated products were noticeably dissimilar from one another, apart from T_B_ (5% GA & 5% MD). T_D_ (10% MD) demonstrated the maximum a_w_ value of 0.450 which was corroborating with previous study carried out by Gurak et al. [42] who found that a_w_ of grape fruit drink microencapsulated by the means of maltodextrin utilizing lyophilization technique was 0.430.

Various factors that determine the solubility of the microencapsulated powdered products includes: the feed composition and particle size. The selection of the wall material is very important, not only for the solubility itself but also to the crystalline state that ultimately bestowed to the dried powders [43]. The aqueous solubility for all lyophilized treatments was ranged from 84.06% to 92.31% as illustrated in Figure 1C. The solubility of the final product possibly not only associated with solubility prospective of microencapsulating wall material but also on attainted particle size in final desirable product; if particle size would be minute, it would ultimately provide the better surface area’s availability for the hydration process [44,45]. The highest solubility value was obtained for treatment T_D_ (10% MD) that was consistent with previous work. Moreira et al. [20] elaborated the solubility percentage for acerola pomace extract ranged from 90.97 to 96.92%, using MD and tree’s gum of cashew apple as microencapsulating wall materials.

The hygroscopicity values for all microencapsulated powder products by the means of freeze-dying method were depicted in Figure 1D. These were ranging from 11.92% to 14.35%, representing a lesser amount of hygroscopicity values for powder products; hence assisted the protection of antioxidant components. The findings of current work have much resemblance with preceding work, utilizing related sort of microencapsulating wall materials. Some renowned investigators reported the hygroscopicity of microencapsulated products made up from bark extract of jaboticaba tree using MD and GA as wall material of 17.75%. The lyophilized powdered products demonstrated the lesser hygroscopic values, regardless the presence of higher contents of moisture [15]. The aforesaid behavior was also reported by Khazaei, et al. [46]. The lower values of hygroscopicity for the all lyophilized products mainly attributed to the bigger particle size, since the bigger the particle size, the lesser the uncovered surface area, therefore low down the water absorption [16,47].

The stability of microencapsulated powdered products for the period of storage was principally determined by glass transition temperatures (T_g_), the lower the T_g_ resulting in lower the stability of final product and vice versa. The glass transition temperatures of all lyophilized products were of 15.86 to 45.0 °C in range (Figure 1E). Amongst all lyophilized microencapsulated products, the T_C_ represented the highest glass transition temperature (45.0 °C), proving maximum stability. Furthermore, other treatments also showed significant values for T_g_ except T_D_. The glass transition temperature has been influenced by diverse factors, including moisture contents, chemical configuration and molecular mass of subjected matter [48]. Adhikari et al., 2004 found the lower transition temperatures of fruit drinks/extract were mainly due to the existence of elevated quantity of low molecular weight organic acids and polysaccharides [49]. Additionally, integration of microencapsulating agents in extracts has much predisposed on glass transition temperatures which varied according to molecular weight of encapsulating material; increase in molecular weight of wall material resulting the increase in final T_g_ of the product. The results of our current work were corroborated with earlier findings [50,51,52]. The lyophilized microencapsulated product obtained from treatment D (T_D_) represented the lower T_g_ because of lower molecular weight of MD. Moreover, this behavior was not noticed in T_C_ (10% GA), T_A_ (10% GA & 5% GE) and T_B_ (10% GA & 5% MD) due to the existence of uppermost molecular weight of GA in the term of quality and quality of wall material.

### 4.3. Bioavailable TP Contents

TP bioavailability ratios, articulated in the term of percentage, were computed by using the equation as followed:

$$Bav\left(\%\right)=\frac{\left[\mathrm{TP}\right]\mathrm{Dialyzable}}{\left[\mathrm{TP}\right]\;\mathrm{Total}}\times 100$$

where, *Bav* (%) represented the percentage (%) for TP bioavailability, whereas [TP] Total and [TP] Dialyzable demonstrated TP concentrations after the PLE extraction method and in vitro digestion procedure respectively.

Figure 1F depicted the bioavailability ratio (%) for all freeze-dried microencapsulated products. Treatment T_A_ and T_C_ demonstrated the excellent bioavailability ratios (%) i.e., 57.25 and 54.64% respectively; there was no significant difference in T_B_ and T_D_. Furthermore, no research has yet been conducted on in vitro dialyzability analysis of aforesaid microencapsulated PHF products.

### 4.4. α-Amylase & α-Glucosidase Inhibition

Type-II DM an outcome of insulin resistance is a metabolic disease that, according to the latest data for the World Health Organization in 2014, impinges on 9% of the world’s population, both in developed and developing countries, and directly caused 1.5 million deaths in that single year [53,54]. In order to hamper the side effects of type-II DM, insulin injection and usage of anti-hyperglycaemic substances are two key conventional approaches. The management of the blood sugar level is effective and novel approach to overcome the diabetes mellitus and related complications. Inhibitors of carbohydrate hydrolyzing enzymes (i.e., α-amylase and α-glycosidase) have been practically valuable as oral hypoglycemic drugs and regarded as a reliable indicator for the efficacy of therapeutic agents [55,56]. Several α-amylase inhibitors including acarbose, miglitol and voglibose are clinically useful to treat diabetes but these are expensive and have considerable clinical side effects. Medicinal plants have great potential to retard the absorption of glucose by inhibiting the saccharides hydrolyzing enzymes [57,58,59,60].

There was an attempt to explore the remarkable drugs from medicinal plants featured with elevated potency and less adverse effects than existing drugs [61,62]. Therefore, screening and isolation of inhibitors from plants for these enzymes are escalating.

**Figure 2:**
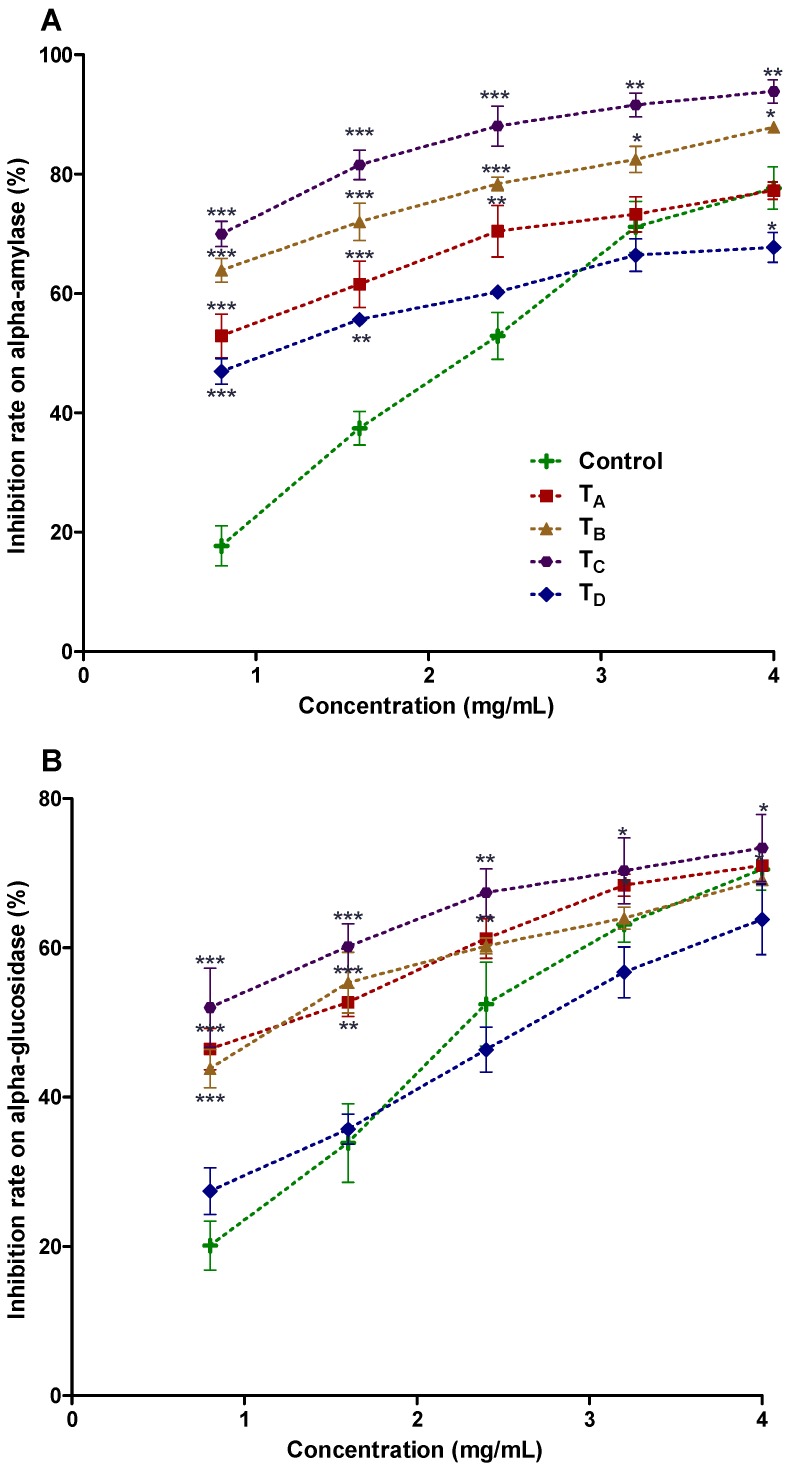
(**A**,**B**) α-amylase and α-glucosidase inhibition activities of PHF extract microencapsulated with GA, GE, MD and their combinations by freeze-drying method. Control: Acarbose; Treatment A (T_A_): Freeze-dried, with 5% GA & 5% GE; Treatment B (T_B_): Freeze-dried, with 5% GA & 5% MD; Treatment C (T_C_): Freeze-dried, with 10% GA; Treatment D (T_D_): Freeze-dried, with 10% MD.

In the aforementioned context, our microencapsulated polyphenolic enriched powders were investigated for α-amylase and α-glycosidase inhibition as shown in Figure 2A,B. Diverse classes of polyphenolic compounds in the current PHF extract were detected likewise: flavonoids, alkaloids, terpenoids, lignans, glycerophospholipid, prenol lipids and their derivatives (detailed in Section 4.7), which eventually may be considered for anti-diabetic potential of microencapsulated powders of current study. The treatment T_C_ (10% GA) demonstrated the highest inhibition at concentration of 4 mg/mL, for α-amylase (93.33 ± 2.65, with IC_50_ value 1.47 mg/mL ± 0.57) and α-glucosidase (73.39 ± 1.66 with IC_50_ value 2.03 ± 0.45 mg/mL), representing highest anti-diabetic potential. Previously, none of investigation has yet been carried out on lyophilized aforementioned microencapsulated PHF products. Additionally, there is no report on microencapsulation of polyphenol enriched extract from PHF and their characterization for anti-diabetic potential purposes, which eventually facilitate to take decision for commercialization of microencapsulated products i.e., polyphenols enriched nutrient supplement.

### 4.5. Size Distribution and Morphology of Microencapsulated Powders

**Figure 3:**
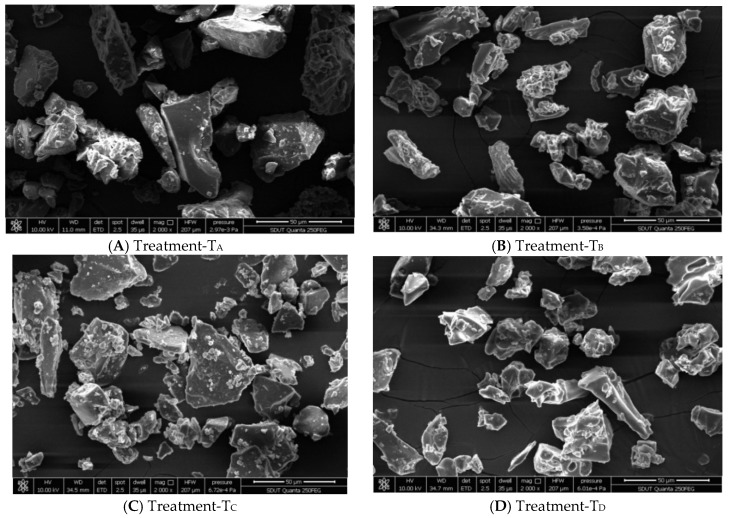
(**A**–**D**) Micrographs of PHF extract microencapsulated with GA, GE, MD and their combinations by freeze-drying method. (**A**) Treatment-T_A_: Freeze-dried, with 5% GA & 5% GE; (**B**) Treatment-T_B_: Freeze-dried, with 5% GA & 5% MD; (**C**) Treatment-T_C_: Freeze-dried, with 10% GA; (**D**) Treatment-T_D_: Freeze-dried, with 10% MD.

Different polymers exhibited particular protection capacity, so the evaluation of microencapsulated products is very crucial. This aforesaid capacity elaborated the extent of micro-pores and reliability of encapsulated micro-particles [63]. The structural analysis of the encapsulated products from the lyophilization methodology was conducted by the means of scanning electron microscope (Quanta 250 EFI). Comparison of the images illustrated the noticeable variation in term of particle structure and size allocation amongst the different microencapsulated products and their combination attained after lyophilization. Figure 3A–D demonstrated the morphology of all freeze-dried microencapsulated products. As can be seen all lyophilized products presented the irregular shape like broken glass with appreciable proportion of pores on surface. The outcome of current investigation has agreement with the recent work explored by Kuch and Norena [64]. These authors studied on morphological aspects of lyophilized products, made up from the peel of grapes and pomace of *Averrhoa carambola* and presented the final product as porous, uneven and brittle conformation; furthermore they also described the reason behind the high porosity of lyophilized products as development of ice crystals had happen in material which as a result retarded the breakdown of final configuration and hence less change in volume occurred.

There was a direct association between span value and dispersal of particle size, the lesser span value demonstrating a uniform distribution of micro-particles [65]. The size of micro-particles from the final products was in the range of 18.08 to 391.30 μm. T_A_ explored the higher particle size (more than 287 μm), whereas T_D_ showed the lowest one (Table 2). Our current work is consistent with prior investigation, examined by other authors [57] who found that the particle size of microencapsulated product via freeze-dying method reached up to 300 μm. The bigger particle dimension of lyophilized products was mainly attributed to rapid freezing and less availability of force to crush the freeze drop during lyophilization [66,67]. Moreover, particle size was also influenced by crushing procedure which was generally accustomed for size reduction after lyophilization.

### 4.6. Acute Toxicity

No toxic effects and mortality were observed at a dose of 2000 mg/kg by gavage. Consequently, microencapsulated products of PHF extract were regarded as safe for consumption.

### 4.7. Bioactive Compounds from LC-ESI-QTOF-MS Analysis

**Figure 4:**
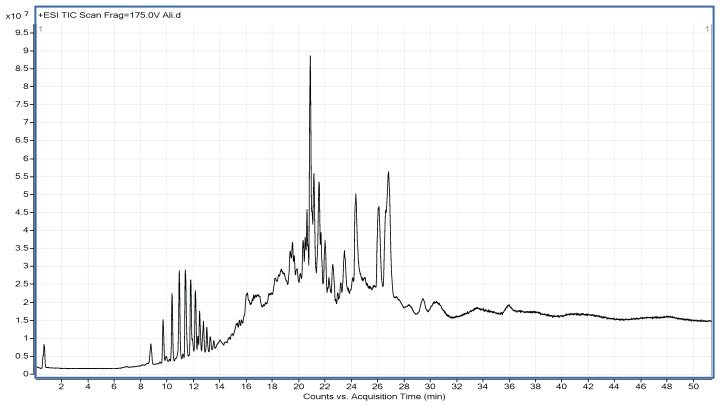
Chromatogram of the Ethanolic extract derived from freeze dried powder of PHF.

The ethanolic extract of freeze dried fine powder of PHF was a multifaceted mixture of compounds. Figure 4 characterized the chromatogram of said ethanolic extract. The bioactive compounds were recognized by means of the comparing retention times (RT) and MS/MS spectra granted by QTOF-MS with those of valid standards wherever obtainable and via elucidation of MS and MS/MS spectra from QTOF-MS merged with data available in literature. MS data of identified compounds has been recapitulated in the Table 3 including calculated *m*/*z* for molecular formulas provided, main fragment obtained by MS/MS, error and proposed compound for each peak. Diverse classes of polyphenolic compounds have been discovered in the ethanolic extract of PHF. Annotated compounds represented the diverse classes includes flavonoids, alkaloids, terpenoids, lignans, glycerophospholipid and prenol lipids.

Peaks 4, 8, 9, 10, 11, 12, 14, 15, 17, 18, 19, 28, 32, 36, 39, 41, 44 and 45 represented different flavonoid compounds and their derivatives which possess diverse previously proven biological activities i.e., anti-inflammatory, anti-nociceptive, anti-oxidative, anti-dengue, anti-malarial [68,69,70,71,72,73,74]. Among them 3 bioactive compounds (peak 9, 18 and 39) were classified as 6-prenylated flavones (i.e., flavones that features a C5-isoprenoid substituent at the 6-position). These bioactive compounds are insoluble in aqueous solution and designated as a faintly acidic compound. These compounds previously found in fruits, peas and pulses and considered to be flavonoid lipid molecules. While some compounds (peak 17, 41, 44 and 45) belong to sub class flavonoids glycosides likewise; quercetagetin 7-glucoside (compound 17, *m*/*z* 481.2572 [M + H]) and quercetin 3-(6″-malonylglucoside)-7-glucoside (Compound 41, *m*/*z* 713.5121 [M + H]) were recognized as flavonoid-7-o-glycosides. These are phenolic compounds containing a flavonoid moiety which is *O*-glycosidically linked to carbohydrate moiety at the C 7-position. These derivatives of flavonoids have priory proved strong antioxidative, anticancer, neuro-protective, anti-inflammatory, diuretic, hypoglycemic and anti-hepatitis activities [75,76]. Moreover, catalpol *m*/*z* 363.195 [M + H] (compound 15) demonstrated a variety of biological activities including anti-cancer, neuro-protective, anti-inflammatory, diuretic, hypoglycemic and anti-hepatitis virus effects. Previous studies have also provided some clues that catalpol can affect energy metabolism through increasing mitochondrial biogenesis, enhancing endogenous antioxidant enzymatic activities and inhibiting free radical generation ultimately attenuates oxidative stress [77].

Mesaconitine (peak 33, *m*/*z* 631.4345 [M + H]) and antherospermidine (peak 3, *m*/*z* 305.1541 [M + H]^+^) were the member of group named alkaloids, later have a structure that contains an aminoethylphenanthrene moiety. Atherosperminine has been cited to be in fruits and bark of *Cryptocarya nigra* (Lauraceae) and have strong antioxidant, anti-mlarial and anti-microbial activities [67]. Steviol, *m*/*z* 31 9.1329 [M + H] designated as (peak 2) in our list of metabolites, is diterpene alkaloids with a structure that is based on the kaurane skeleton. It possesses a [3, 2, 1]-bicyclic ring system with C15-C16 bridge connected to C13, forming the five-member ring D. This compound was excessively found in different sorts of fruits and primarily responsible for the sweet taste of stevia leaves. This compound is considered safe for human consumption and was approved as a food additive by the Food and Drugs Administration (FDA) and European Food Safety Authority (EFSA) and helped to reduce the oxidative stress [78]. In addition, the peak 6 is of zanthobisquinolone, *m*/*z* 363.1589 [M + H] and peak 7 is of murrayazolinol, *m*/*z* 349.1795 [M + H] belongs to the class quionlines and their derivatives, also alkaloid in nature. These are usually present in herbs, spices and some fruits [79,80,81]. Various anti-malarial, anti-parasitic, anti-bacterial and anti-viral drugs do contain a major constitute of aforesaid bioactive compound [82].

Besides flavonoid and alkaloids, these are also some compounds which have appreciable share, belong to class prenol lipids (peak 20, 23, 37 and 42). Amongst, ubiquinol 8 (peak 37, *m*/*z* 729.5073 [M + H]*^+^* belongs to organic compounds known as polyprenyl quinols. It is the reduced configuration of ubiquinone-8. It plays a function as an electron transporter in mitochondrial membrane, where it carries two electrons from either complex I (i.e., NADH dehydrogenase) or complex II (i.e., succinate-ubiquinone reductase) to complex III, whilst, compound 42, named phytoene fragmented at *m*/*z* 545.1143 [M + H] is member of class regarded as carotenes and further belongs to family carotenoids. These are unsaturated hydrocarbons comprising of eight repeated isoprene units. They have also previously proven antioxidant, anti-cancer activity and facilitate to reduce the complications [83].

Amongst the known natural bioactive compounds, terpenoids are considered to be of approximately 60%. Plant terpenoids are extensively used for their aromatic qualities and play a role in traditional herbal remedies, for instance; *Euphorbia* diterpenoids 3 (peak 22) possesses a variety of different core frameworks and exhibit a diverse array of beneficial activities, including anti-tumor, anti-inflammation, and immune-modulatory features and regarded as excellent source in term of scientific attraction [84]. On the other hand, Buddledin A (peak 25) a sesquiterpenoid based on a humulane skeleton, displaying selective anti-fungal activity against dermatophytes [85], while 3-*O*-*cis*-coumaroylmaslinic acid (peak 29) have ability to attenuate oxidative stress.

Lignans were usually found in fruits and have proved strong anti-cancer and antioxidant activities. Among them, compound 16 (secoisolariciresinol) and compound 13 belong to class dibenzylbutane lignans and furanoid lignans respectively. It was present in a number of food items such as American butterfish, Brazil nut, fireweed, and oriental wheat [86,87]. Besides these, peak 5 and 34 represented phloretin and xanthoangelol respectively. These compounds showed various biological activities i.e., anti-tumor and anti-metastatic features [88,89].

Other detected compounds which were not discussed in detailed such as peak 21, 24, 26, 27, 30, 31 35, are intermediate products of either metabolism or biosynthesis of amino acids, (phosphor or/and sphingo) lipids or others. For instance, metabolite 24th represented a phosphatidylethanolamine, is an anchor protein, produced as an intermediate in gycosylphosphatidylinositol (GPI) anchor biosynthesis pathway, while compound 30 and compound 35 are the phosphatidic acids, produced in glycerolipid biosynthesis. The existences of such compounds are mainly attributed to the seeds of *Hygrophila spinosa* T. Anders [90]. The 26th peak recognized as phytosphingosine, *m*/*z* 318.2974 [M + H], is an intermediate compound synthesized between dihrdro-shingosine and phyto-ceramide in shingophospholipid metabolism. Phospholipids have diverse functions in varied processes of cell i.e., apoptosis, cell propagation, cell to cell interaction, differentiation etc. Furthermore, phytosphingosine is naturally occurring sphingoid bases, fungi and plants are the rich source of phytosphingosine. It is structurally similar to sphingosine; phytosphingosine possesses a hydroxyl group at C-4 of the sphingoid long-chain base. Phyto-sphingosine induces apoptotic cell death in human cancer cells by direct activation of caspase 8, and by mitochondrial translocation of Bax and subsequent release of cytochrome C into cytoplasm, providing a potential mechanism for the anti-cancer activity of phytosphingosine. The metabolite 01, *m*/*z* 261.128 [M + H] have been referred to caffeic acid 4-sulfate (polyphenol) belongs to a class cinnamic acids and their derivatives. Hydroxycinnamic acids are compounds containing a cinnamic acid where the benzene ring is hydroxylated. It is one of the most representative phenolic acids in fruits and vegetables which have excellent antioxidative potential and anti-carcinogenic activity [90,91,92,93]_._

As can been concluded that the current PHF is the mixture of previously proven [21,22,23,24] health promoting herbs’ parts, so diversity and abundance of such detected antioxidant substances/metabolites not only made sense but also verify the outcomes. Taking together, this is the first study which exploited the metabolite profiling of said PHF enriched with antioxidants and their evaluation for bioavailability and anti-diabetic potential after encapsulation.

## 5. Conclusions

In the current study, PHF polyphenolic extract was microencapsulated by utilizing GA, GE, and MD as encapsulating wall materials, due to which resulting microcapsules found to have withholding capacity of TPC more than 85% except T_D_ (68.22%), while conserving range of TFC and TCT were found near to 60% except T_A_. Elevated antioxidant activity was also revealed for T_B_ and T_C_ and reasonable for T_A_ and T_D,_ representing noteworthy and positive correlation of antioxidant assays to all aforementioned antioxidant components. Taking all results into consideration, T_B_ (5% GA & 5% MD) and T_C_ (10% GA) showed the best performance attributable with respect to the higher preservation of antioxidant components and antioxidant activity by means of DPPH and β-carotene assays and significant for an ABTS^+^ radical scavenging activity, augmented by low contents of moisture, water activity (a_w_), particle dimension and elevated solubility, hygroscopicity and T_g._ Additionally, the aforementioned treatments also demonstrated the excellent morphological features with asymmetrical (irregular) micro-particle structures, depicted lower prevalence of coarseness and crankiness. Moreover, T_B_, T_C_ and T_A_ also characterized the highest anti-diabetic potential by reason of their significant inhibition rate for α-amylase and α-glucosidase. In the context of bioavailability, T_B_ and T_C_ also demonstrated the excellent bioavailability ratios (%) (i.e., more than 50% & 40% respectively). The bioavailability data revealed that microencapsulation of PHF (especially with T_C_ and T_B_) can improve the bioavailability of pH and thermo-labile bioactive compounds at intestinal level which is a major site for absorption of bioactive compounds. In addition, no mice proved any toxicity sign at a dose of 2000 mg/kg by gavage for any treatment. In the conclusive manner, we recommended the T_B_ and T_C_ as result of their incredible capability for preserving antioxidant components to its usage in nutraceutical and functional products while masking the undesirable flavor distinctiveness of herbs/herbal extracts.

## Figures and Tables

**Table 1:** Antioxidant components and antioxidant activities of PHF extracts microencapsulated with gum arabic (GA), gelatin (GE), maltodextrin (MD) and their combinations by Freeze-drying method.

Treatments	TPC ^1^	TFC ^2^	TCT ^3^	DPPH ^4^	Beta-carotene ^5^	ABTS ^6^
Control	26.72 ± 0.61 ^a^	6.848 ± 0.05 ^a^	15.72 ± 0.3 ^a^	133.3 ± 1.79 ^a^	83.39 ± 0.79 ^a^	3.687 ± 0.03 ^a^
T_A_	22.89 ± 0.41 ^b^	2.760 ± 0.03 ^e^	9.383 ± 0.16 ^d^	74.73 ± 4.6 ^d,e^	64.71 ± 0.64 ^d,e^	3.197 ± 0.95 ^b^
T_B_	24.26 ± 0.085 ^a^	4.183 ± 0.07 ^c^	10.10 ± 0.13 ^c^	85.0 ± 0.5 ^b^	78.34 ± 0.51 ^b^	2.777 ± 0.125 ^c^
T_C_	25.26 ± 0.22 ^a^	5.233 ± 0.15 ^b^	12.46 ± 0.021 ^b^	78.11 ± 1.67 ^c^	75.40 ± 0.88 ^c^	2.733 ± 0.06 ^c^
T_D_	18.27 ± 0.15 ^c^	3.817 ± 0.03 ^d^	9.383 ± 0.07 ^d^	51.52 ± 0.72 ^f^	65.72 ± 0.92 ^d,e^	2.285 ± 0.072 ^d,e^

**Note: **Results displayed are a representation of triplicate quantifications per extract. T_A_: Freeze-dried, with 5% GA and 5% GE; T_B_: Freeze-dried, with 5% GA and 5% MD; T_C_: Freeze-dried, with 10% GA; T_D_: Freeze-dried, with 10%. **^1 ^**Total phenolic contents (TPC) expressed as mg gallic acid equivalents (GAE) per g of dry extract; **^2 ^**Flavonoid content expressed as mg quercetin equivalents (QE) per g of dry extract; **^3 ^**Total condensed tannin content based on calibration curve of (+)-catechin, expressed as mg catechin equivalents (CE) per g of dry extract. **^4 ^**DPPH expressed as µmol/g sample on dry basis; **^5 ^**β-carotene of extracts (5 mg/mL) based on percent bleaching inhibition. **^6 ^**EC_50_ (mg/mL) is representative of the effective concentration at which 50% of ABTS^+^ radicals were scavenged. The Dunnett’s test was to evaluate the significance with confidence level was set to 95%; different letters within the same column indicate significant differences (*p *< 0.05).

**Table 2:** Average diameter and particle size distribution (Span) of the PHF extract microencapsulated with GA, GE, MD and their combinations by freeze-drying method.

Treatments	Average Diameter (µm)	Span
T_A_	151.13	1.74
T_B_	76.15	1.21
T_C_	92.79	2.88
T_D_	18.95	1.52

Treatment A (T_A_): Freeze-dried, with 5% GA and 5% GE; Treatment B (T_B_): Freeze-dried, with 5% GA and 5% MD; Treatment C (T_C_): Freeze-dried, with 10% GA; Treatment D (T_D_): Freeze-dried, with 10% MD.

**Table 3:** Bioactive Compounds identified in Ethanolic Extract of PHF.

Peak No.	RT (min)	Assigned Compound Name	Elemental Composition	*m*/*z* [M + H]^+^	Difference (mDa)
1	8.773	Caffeic acid 4-sulfate	C_9_H_8_O_7_S	261.128	0.71
2	9.458	Steviol	C_20_H_30_O_3_	319.1329	−0.92
3	9.708	Antherospermidine	C_18_H_11_NO_4_	305.1541	0.15
4	9.904	Eriodictyol	C_15_H_12_O_6_	289.1231	−0.14
5	9.95	Phloretin	C_15_H_14_O_5_	275.1077	1.04
6	10.177	Zanthobisquinolone	C_21_H_18_N_2_O_4_	363.1589	0.57
7	10.384	Murrayazolinol	C_23_H_25_NO_2_	349.1795	0.72
8	10.639	Patuletin	C_16_H_12_O_8_	333.1488	0.34
9	10.746	Albanin d	C_25_H_26_O_5_	407.1849	0.84
10	10.834	3,5,8,3′,4′,5′-Hexahydroxyflavone	C_15_H_10_O_8_	319.1329	0.31
11	10.918	Myricetin	C_15_H_10_O_8_	319.1692	0.3
12	10.936	Dehydroneotenone	C_19_H_12_O_6_	393.2055	0.77
13	11.219	Carissanol	C_20_H_24_O_7_	377.1746	0.86
14	11.4	Epigallocatechin 3-*O*-cinnamate	C_24_H_20_O_8_	437.2313	−0.13
15	11.479	Catalpol	C_15_H_22_O_10_	363.195	0.8
16	11.528	Secoisolariciresinol	C_20_H_26_O_6_	363.1589	0.46
17	11.805	Quercetagetin 7-glucoside	C_21_H_20_O_13_	481.2572	−0.02
18	11.945	Cajaflavanone	C_25_H_26_O_5_	407.2208	−0.17
19	12.163	Barbatoflavan	C_24_H_28_O_13_	525.2828	0.9
20	12.348	Celastrol	C_29_H_38_O_4_	451.2469	0.04
21	12.484	6-Gingerol	C_17_H_26_O_4_	296.1487	−1.92
22	12.77	*Euphorbia* diterpenoid 3	C_33_H_40_O_11_	613.3348	0.89
23	13.017	2-Hexaprenyl-6-methoxyphenol	C_37_H_56_O_2_	534.3435	0.67
24	13.278	PE(P-16:0/18:2(9Z,12Z))	C_39_H_74_NO_7_P	701.3867	−1.1
25	13.569	Buddliedin A	C_17_H_24_O_3_	277.1385	−0.24
26	16.104	Phytosphingosine	C_18_H_39_NO_3_	318.2974	−0.04
27	17.059	1-Eicosenee	C_20_H_40_	282.2015	0.84
28	19.53	Tectorigenin	C_16_H_12_O_6_	301.1391	0.18
29	20.643	3-*O*-*cis*-Coumaroylmaslinic acid	C_39_H_54_O_6_	619.3973	−0.4
30	20.884	PA(18:3(6Z,9Z,12Z)/20:3(8Z,11Z,14Z))	C_41_H_69_O_8_P	721.4644	0.32
31	21.141	Eurysterol A sulfonic acid	C_27_H_46_O_7_S	515.3518	0.33
32	21.548	Citflavanone	C_20_H_18_O_5_	338.3391	1.25
33	21.698	Mesaconitine	C_33_H_45_NO_11_	631.4345	−0.17
34	22.01	Xanthoangelol	C_25_H_28_O_4_	393.294	0.44
35	22.385	PA(15:0/22:4(7Z,10Z,13Z,16Z))	C_40_H_71_O_8_P	711.4757	0.56
36	22.589	2′,5,6-trimethoxyflavone	C_18_H_16_O_5_	312.3236	1.42
37	22.613	Ubiquinol-8	C_49_H_76_O_4_	729.5073	0.38
38	22.646	Epicalyxin J	C_42_H_38_O_9_	686.4852	0.14
39	22.967	Luteone	C_20_H_18_O_6_	354.37	1.64
40	23.177	Luteolin 4′-sulfate	C_15_H_10_O_9_S	366.3702	1.43
41	23.524	Quercetin 3-(6″-malonylglucoside)-7-glucoside	C_30_H_32_O_20_	713.5121	0.64
42	24.348	Phytoene	C_40_H_64_	545.1143	0.39
43	26.083	Epigallocatechin 3,3′,-di-*O*-gallate	C_29_H_22_O_15_	610.1796	0.62
44	26.819	Kaempferol 3-(2″,3″-diacetyl-4″-p-coumaroylrhamnoside)	C_34_H_30_O_14_	663.4496	0.47
45	29.446	Delphinidin 3-(6″-malonyl-glucoside)	C_24_H_23_O_15_	684.1982	0.21

## References

[1] Abbas M., Saeed F., Anjum F.M., Afzaal M., Tufail T., Bashir M.S., Ishtiaq A., Hussain S., Suleria H.A.R. (2016). Natural polyphenols: An overview. Int. J. Food Propert..

[2] Hameed A., Hussain S.A., Yang J., Ijaz M.U., Liu Q., Suleria H.A.R., Song Y. (2017). Antioxidants Potential of the Filamentous Fungi (*Mucor circinelloides*). Nutrients.

[3] Suleria H.A., Butt M.S., Anjum F.M., Saeed F., Khalid N. (2015). Onion: Nature protection against physiological threats. Crit. Rev. Food Sci. Nutr..

[4] Hurrle S., Hsu W.H. (2017). The etiology of oxidative stress in insulin resistance *Biomed*. J..

[5] Yousaf S., Butt M.S., Suleria H.A., Iqbal M.J. (2014). The role of green tea extract and powder in mitigating metabolic syndromes with special reference to hyperglycemia and hypercholesterolemia. Food funct..

[6] Ameer K., Shahbaz H.M., Kwon J.H. (2017). Green extraction methods for polyphenols from plant matrices and their byproducts: A review. Compr. Rev. Food Sci. Food Saf..

[7] Mojzer E.B., Knez Hrnčič M., Škerget M., Knez Ž., Bren U. (2016). Polyphenols: Extraction methods, antioxidative action, bioavailability and anticarcinogenic effects. Molecules.

[8] Spinella M. (2002). The importance of pharmacological synergy in psychoactive herbal medicines. Altern. Med. Rev..

[9] Chorgade M.S. (2007). Drug Discovery and Development.

[10] Pole S. (2013). Ayurvedic Medicine: The Principles of Traditional Practice.

[11] Fang Z., Bhandari B. (2011). Effect of spray drying and storage on the stability of bayberry polyphenols. Food Chem..

[12] Garber A.J., Karlsson F.O. (2001). Treatment of dyslipidemia in diabetes. Endocrinol. Metab. Clin..

[13] Lee B.R., Lee Y.P., Kim D.W., Song H.Y., Yoo K.Y., Won M.H., Kang T.C., Lee K.J., Kim K.H., Joo J.H. (2010). Amelioration of streptozotocin- induced diabetes by *Agrocybe chaxingu* polysaccharide. Mol. Cells.

[14] Hu S.H., Wang J.C., Lien J.L., Liaw E.T., Lee M.Y. (2006). Antihyperglycemic effect of polysaccharide from fermented broth of *Pleurotus citrinopileatus*. Appl. Microbiol. Biotechnol..

[15] Silva P.I., Stringheta P.C., Teófilo R.F., de Oliveira I.R.N. (2013). Parameter optimization for spray-drying microencapsulation of jaboticaba (*Myrciaria jaboticaba*) peel extracts using simultaneous analysis of responses. J. Food Eng..

[16] Tonon R.V., Brabet C., Pallet D., Brat P., Hubinger M.D. (2009). Physicochemical and morphological characterization of açai (*Euterpe oleraceae* Mart.) powder produced with different carrier agents. Int. J. Food Sci. Technol..

[17] Moreira G.E.G., de Azeredo H.M.C., de Medeiros M.d.F.D., de Brito E.S., de Souza A.C.R. (2010). Ascorbic acid and anthocyanin retention during spray drying of acerola pomace extract. J. Food Process. Preserv..

[18] Tonon R.V., Brabet C., Hubinger M.D. (2008). Influence of process conditions on the physicochemical properties of acai (*Euterpe oleraceae* Mart.) powder produced by spray drying. J. Food Eng..

[19] Mathiowitz E., Mathiowitz E. (1999). Microencapsulation. Encyclopedia of controlled drug delivery.

[20] Mahdavi S.A., Jafari S.M., Assadpoor E., Dehnad D. (2016). Microencapsulation optimization of natural anthocyanins with maltodextrin, gum arabic and gelatin. Int. J. Biol. Macromol..

[21] Varghese C.P. (2013). Antioxidant and anti-inflammatory activity of *Eurycoma longifolia* Jack, A traditional medicinal plant in Malaysia. Int. J. Pharm. Sci. Nanotechnol..

[22] Shahzad M., Shabbir A., Wojcikowski K., Wohlmuth H., Gobe G.C. (2016). The Antioxidant Effects of Radix Astragali (*Astragalus membranaceus* and related Species) in Protecting Tissues from Injury and Disease. Curr. Drug Target..

[23] Patra A., Jha S., Murthy P.N. (2009). Phytochemical and pharmacological potential of *Hygrophila spinosa* T. Anders. Pharmacol. Rev..

[24] Sharma G., Kumar M. (2012). Antioxidant and modulatory role of *Chlorophytum borivilianum* against arsenic induced testicular impairment. J. Eniron. Sci..

[25] Suleria H.A.R., Butt M.S., Anjum F.M., Saeed F., Batool R., Ahmad A.N. (2012). Aqueous garlic extract and its phytochemical profile; special reference to antioxidant status. Int. J. Food Sci. Nutr..

[26] Brand-Williams W., Cuvelier M.E., Berset C. (1995). Use of free radical method to evaluate antioxidant activity. LWT Food Sci. Technol..

[27] AOAC (1990). Official Methods of Analysis of the Association of Official Analytical Chemists.

[28] Cano-Chauca M., Stringheta P.C., Ramos A.M., Cal-Vidal J. (2005). Effect of the carriers on the microstructure of mango powder obtained by spray drying and its functional characterization. Innov. Food Sci. Emerg. Technol..

[29] Caparino O.A., Tang J., Nindo C.I., Sablani S.S., Powers J.R., Fellman J.K. (2012). Effect of drying methods on the physical properties and microstructures of mango (Philippine ‘Carabao’ var.) powder. J. Food Eng..

[30] Young-In K., Apostolidis E., Shetty K. (2008). Inhibitory potential of wine and tea against α-amylase and α-glucosidase for management of hyperglycemia linked to type 2 diabetes. J. Food Biochem..

[31] Apostolidis E., Kwon Y.I., Shetty K. (2007). Inhibitory potential of herb, fruit, and funga-enriched cheese against key enzymes linked to type 2 diabetes and hypertension. Innov. Food Sci. Emerg. Technol..

[32] Moreda-Pineiro J., Herbello-Hermelo P., Domínguez-González R., Bermejo-Barrera P., Moreda-Piñeiro A. (2016). Bioavailability assessment of essential and toxic metals in edible nuts and seeds. Food Chem..

[33] OECD (1995). OECD Guidelines for the Testing of Chemicals, Repeated Dose 28-Day Oral Toxicity Study in Rodents.

[34] Bringmann G., Kajahn I., Neusüß C., Pelzing M., Laug S., Unger M., Holzgrabe U. (2005). Analysis of the glucosinolate pattern of Arabidopsis thaliana seeds by capillary zone electrophoresis coupled to electrospray ionization-mass spectrometry. Electrophoresis.

[35] Golde P.H., van der Westelaken M., Bouma B.N., van de Wiel A. (2004). Characteristics of piraltin, a polyphenol concentrate, produced by freeze-drying of red wine. Life Sci..

[36] Silva P.B., Duarte C.R., Barrozo M.A.S. (2016). Dehydration of acerola (*Malpighia emarginata* D.C.) residue in a new designed rotary dryer: Effect of process variables on main bioactive compounds. Food Bioprod. Process..

[37] Ramírez M.J., Giraldo G.I., Orrego C.E. (2015). Modeling and stability of polyphenol in spray-dried and freeze-dried fruit encapsulates. Powder Technol..

[38] Burin V.M., Rossa P.N., Ferreira-Lima N.E., Hillmann M.C., Boirdignon-Luiz M.T. (2011). Anthocyanins: Optimization of extraction from Cabernet Sauvignon grapes, microencapsulation and stability in soft drink. Int. J. Food Sci. Technol..

[39] Souza V.B., Thomazini M., de Carvalho Balieiro J.C., Fávaro-Trindade C.S. (2015). Effect of spray drying on the physicochemical properties and color stability of the powdered pigment obtained from vinification byproducts of the Bordo grape (*Vitis labrusca*). Food Bioprod. Process..

[40] Franceschinis L., Salvatori D.M., Sosa N., Schebor C. (2014). Physical and functional properties of blackberry freeze- and spray-dried powders. Dry. Technol..

[41] Ezhilarasi P.N., Indrani D., Jena B.S., Anandharamakrishnan C. (2013). Freeze drying technique for microencapsulation of Garcinia fruit extract and its effect on Bread quality. J. Food Eng..

[42] Gurak P.D., Cabral L.M.C., Rocha-Leao M.H. (2013). Production of grape juice powder obtained by freeze-drying after concentration by reverse osmosis. Braz. Arch. Biol. Technol..

[43] Cortes-Rojas D.F., Souza C.R.F., Oliveira W.P. (2015). Optimization of spray drying conditions for production of *Bidens pilosa* L. dried extract. Chem. Eng. Res. Des..

[44] Dag D., Kilercioglu M., Oztop M.H. (2017). Physical and chemical characteristics of encapsulated goldenberry (*Physalis peruviana* L.) juice powder. LWT Food Sci. Technol..

[45] Çam M., Içyer N.C., Erdogan F. (2014). Pomegranate peel phenolics: Microencapsulation, storage stability and potential ingredient for functional food development. LWT Food Sci. Technol..

[46] Khazaei K.M., Jafari S.M., Ghorbani M., Kakhki A.H. (2014). Application of maltodextrin and gum Arabic in microencapsulation of saffron petal’s anthocyanins and evaluating their storage stability and color. Carbohydr. Polym..

[47] Man Y.B.C., Irwandi J., Abdullah W.J.W. (1999). Effect of different types of maltodextrin and drying methods on physico-chemical and sensory properties of encapsulated durian flavor. J. Sci. Food Agric..

[48] Sawale P.D., Patil G.R., Hussain S.A., Singh A.K., Singh R.R.B. (2017). Release Characteristics of Polyphenols from Microencapsulated Terminalia Arjuna Extract: Effects of Simulated Gastric Fluid. Int. J. Food Prop..

[49] Adhikari B., Howes T., Bhandari B.R., Troung V. (2004). Effect of addition of maltodextrin on drying kinetics and stickiness of sugar and acid-rich during convective drying: Experiments and modelling. J. Food Eng..

[50] Mitchell H.L., Rollern S., Jones S.A. (1996). The role of the bulking agent polydextrose in fat replacement. Handbook of Fat Replacers.

[51] Kapoor M.P., Juneja L.R., Cho S.S., Samuel P. (2009). Partially hydrolyzed guar gum dietary fiber. Fiber Ingredients: Food Applications and Health Benefits.

[52] Al-Assaf S., Philips G.O., Williams P.A. (2005). Studies on Acacia exudates gums: Part II. Molecular weight comparison of the Vulgares and Gummiferae series of Acacia gums. Food Hydrocoll..

[53] Kumar P.S., Sudha S. (2012). Evaluation of α amylase and α glucosidase inhibitory properties of selected seaweeds from Gulf of Mannar. Int. Res. J. Pharm..

[54] Lavelli V., Harsha P.S., Ferranti P., Scarafoni A., Iametti S. (2016). Grape skin phenolics as inhibitors of mammalian α-glucosidase and α-amylase effect of food matrix and processing on efficacy. Food Funct..

[55] Kazeem M.I., Ogunbiyi J.V., Ashafa A.O.T. (2000). In vitro Studies on the Inhibition of α-Amylase and α- Glucosidase by Leaf Extracts of *Picrali manitida* (Stapf) Gin H., Rigalleau V. Post-prandial hyperglycemia and Diabetes. Diabete Metab..

[56] Sima A.A., Chakrabarti S. (1992). Long-term suppression of postprandial hyperglycaemia with acarbose retards the development of neuropathies in the BB/W-rat. Diabetologia.

[57] Kwon Y.I., Apostolidis E., Shetty K. (2007). Evaluation of pepper (*Capsicum annuum*) for management of diabetes and hypertension. J. Food Biochem..

[58] Sultan M.T., Buttxs M.S., Qayyum M.M.N., Suleria H.A.R. (2014). Immunity: Plants as effective mediators. Crit. Rev. Food Sci. Nutr..

[59] Kim J.S., Kwon C.S., Son K.H. (2000). Inhibition of α-glucosidase and amylase by Luteolin, a flavonoid. Biosci. Biotechnol. Biochem..

[60] Matsui T., Ogunwande I.A., Abesundara K.J.M., Sumoto K.M. (2006). Anti-hyperglycemicpotential of natural products. Mini Rev. Med. Chem..

[61] Matsuda H., Morikawa T., Yoshikawa M. (2002). Antidiabetogenic constituents from several natural medicines. Pure Appl. Chem..

[62] Ogunwande I.A., Matsui T., Fujise T., Matsumoto K. (2007). 𝛼-Glucosidase inhibitory profile of Nigerian medicinal plants in immobilized assay system. Food Sci. Technol. Res..

[63] Nunes G.L., Boaventura B.C.B., Pinto S.S., Verruck S., Murakami F.S., Prudêncio E.S., Amboni R.D.D.M.C. (2015). Microencapsulation of freeze concentrated Ilex paraguariensis extract by spray drying. J. Food Eng..

[64] Kuck L.S., Norena C.P.Z. (2016). Microencapsulation of grape (*Vitis labrusca* var. Bordo) skin phenolic extract using gum arabic, polydextrose, and partially hydrolyzed guar gum as encapsulating agents. Food Chem..

[65] Kuang S.S., Oliveira J.C., Crean A.M. (2010). Microencapsulation as a tool for incorporating bioactive ingredients into food. Crit. Rev. Food Sci. Nutr..

[66] Chen C., Chi Y.J., Xu W. (2012). Comparisons on the functional properties and antioxidant activity of spray-dried and freeze-dried egg white protein hydrolysate. Food Bioproess Technol..

[67] Saikia S., Mahnot N.K., Mahanta C.L. (2015). Optimization of phenolic extraction from *Averrhoa carambola* pomace by response surface methodology and its microencapsulation by spray and freeze drying. Food Chem..

[68] Eldeen A., Kawashty S.A., Ibrahim L.F., Shabana M.M., El-Negoumy S.I. (2004). Evaluation of antioxidant, anti-inflammatory, and antinociceptive properties of aerial parts of *Vicia sativa* and its flavonoids. J. Nat. Remed..

[69] Tisserant L.P., Hubert J., Lequart M., Borie N., Maurin N., Pilard S., Jeande P., Aziz A., Renault J.-H., Nuzillard J.-M. (2016). 13C NMR and LC-MS Profiling of Stilbenes from Elicited Grapevine Hairy Root Cultures. J. Nat. Prod..

[70] Chobot V., Hadacek F., Bachmann G., Weckwerth W., Kubicova L. (2016). Pro- and Antioxidant Activity of Three Selected Flavan Type Flavonoids: Catechin, Eriodictyol and Taxifolin. Int. J. Mol. Sci..

[71] Hassan S.M., Khalaf M.M., Sadek S.A., Abo-Youssef A.M. (2017). Protective effects of apigenin and myricetin against cisplatin-induced nephrotoxicity in mice. Pharm. Biol..

[72] Cuendet M., Potterat O., Hostettmann K. (2001). Flavonoids and phenylpropanoid derivatives from *Campanula barbata*. Phytochemistry.

[73] Tang L., Ling A.P., Koh R.Y., Chye S.M., Voon K.G. (2008). Screening of anti-dengue activity in methanolic extracts of medicinal plants. BMC Complement. Altern. Med..

[74] Khaomek P., Ichino C., Ishiyama A., Sekiguchi H., Namatame M., Ruangrungsi N., Saifah E., Kiyohara H., Otoguro K., Omura S. (2008). In vitro antimalarial activity of prenylated flavonoids from *Erythrina fusca*. J. Nat. Med..

[75] Saeed F., Ahmad R.S., Arshad M.U., Niaz B., Batool R., Naz R., Suleria H.A.R. (2015). Propolis to Curb Lifestyle Related Disorders: An Overview. Int. J. Food Prop..

[76] Agostino M., De Simone F., Piacente S., Pizza C., Senatore F. (1997). Quercetagetin 6-*O*-β-d-glucopyranoside from *Tagetes mandonii*. Phytochemistry.

[77] Zhang Y., Wang C., Yang Q., Jin Y., Meng Q., Liu Q., Dai Y., Liu Z., Liu K., Sun H. (2017). Catalpol attenuates oxidative stress and promotes autophagy in TNF-a-exposed HAECs by up-regulating AMPK. RSC Adv..

[78] Aceves L., Dublán-García O., López-Martínez L.X., Novoa-Luna K.A., Islas-Flores H., Galar-Martínez M., García-Medina S., Hernández-Navarro M.D., Gómez-Oliván L.M. (2017). Reduction of the Oxidative Stress Status Using Steviol Glycosides in a Fish Model (*Cyprinus carpio*). BioMed Res. Int..

[79] Shmuel Y. (2004). Dictionary of Food Compounds with CD-ROM: Additives, Flavors, and Ingredients.

[80] Cedrón J.C., Gutiérrez D., Flores N., Ravelo Á.G., Estévez-Braun A. (2013). Preparation and antimalarial activity of semisynthetic lycorenine derivatives. Euro. J. Med. Chem..

[81] Buckingham J., Munasingh V.R.N. (2007). Dictionary of Flavonoids.

[82] Palluotto F., Sosic A., Pinato O., Zoidis G., Catto M., Sissi C., Catto B., Carotti A. (2016). Quinolino [3, 4-b] quinoxalines and pyridazino [4, 3-c] quinoline derivatives: Synthesis, inhibition of topoisomerase II α, G-quadruplex binding and cytotoxic properties. Eur. J. Med. Chem..

[83] Fiedor J., Brrda K. (2014). Potential Role of Carotenoids as Antioxidants in Human Health and Disease. Nutrients.

[84] Buckingham J., Baggaley K.H., Roberts A.D., Szabo L.F. Dictionary of Alkaloids, Second Edition with CD-ROM, 2010. https://books.google.com/books?id=mynNBQAAQBAJ.

[85] Mensah A.Y., Houghton P.J., Bloomfield S., Vlietinck A., Vanden Berghe D. (2000). Known and novel terpenes from *Buddleja globosa* displaying selective antifungal activity against dermatophytes. J. Nat. Prod..

[86] Chun H., Yuan Y.V., Kitts D.D. (2007). Antioxidant activities of the flaxseed lignan secoisolariciresinol diglucoside, its aglycone secoisolariciresinol and the mammalian lignans enterodiol and enterolactone in vitro. Food Chem. Toxicol..

[87] Wangteeraprasert R., Lipipun V., Gunaratnam M., Neidle S., Gibbons S., Likhitwitayawuid K. (2012). Bioactive compounds from *Carissa spinarum*. Phytother. Res..

[88] Stangl V., Lorenz M., Ludwig A., Grimbo N., Guether C., Sanad W., Ziemer S., Martus P., Baumann G., Stangl K. (2005). The flavonoid phloretin suppresses stimulated expression of endothelial adhesion molecules and reduces activation of human platelets. J. Nutr..

[89] Sumiyoshi M., Taniguchi M., Baba K., Kimura Y. (2015). Antitumor and antimetastatic actions of xanthoangelol and 4-hydroxyderricin isolated from *Angelica keiskei* roots through the inhibited activation and differentiation of M2 macrophages. Phytomedicine.

[90] Cui L., Decker E.A. (2016). Phospholipids in foods: Pro-oxidants or antioxidants. J. Sci. Food Agric..

[91] Dugasani S., Pichika M.R., Nadarajah V.D., Balijepalli M.K., Tandra S., Korlakunta J.N. (2010). Comparative antioxidant and anti-inflammatory effects of [6]-gingerol, [8]-gingerol, [10]-gingerol and [6]-shogaol. J. Ethnopharmacol..

[92] Park M.T., Kang J.A., Choi J.A., Kang C.M., Kim T.H., Bae S., Kang S., Kim S., Choi W., Cho C.-K. (2003). Phytosphingosine induces apoptotic cell death via caspase 8 activation and Bax translocation in human cancer cells. Clin. Cancer Res..

[93] Tezuka Y., Gewali M.B., Ali M.S., Banskota A.H., Kadota S. (2001). Eleven Novel Diarylheptanoids and Two unusual Diarylheptanoid Derivatives from the Seeds of *Alpinia blepharocalyx*. J. Nat. Prod..

